# Linking High Risk Postpartum Women with a Technology Enabled Health Coaching Program to Reduce Diabetes Risk and Improve Wellbeing: Program Description, Case Studies, and Recommendations for Community Health Coaching Programs

**DOI:** 10.1155/2016/4353956

**Published:** 2016-10-26

**Authors:** Priyanka Athavale, Melanie Thomas, Adriana T. Delgadillo-Duenas, Karen Leong, Adriana Najmabadi, Elizabeth Harleman, Christina Rios, Judy Quan, Catalina Soria, Margaret A. Handley

**Affiliations:** ^1^Department of Epidemiology and Biostatistics, University of California San Francisco, San Francisco, CA, USA; ^2^UCSF Center for Vulnerable Populations, Zuckerberg San Francisco General Hospital, San Francisco, CA 94110, USA; ^3^Department of Psychiatry, UCSF/Zuckerberg San Francisco General Hospital, San Francisco, CA, USA; ^4^Division of General Internal Medicine, UCSF/Zuckerberg San Francisco General Hospital, University of California San Francisco, San Francisco, CA, USA; ^5^Department of Obstetrics and Gynecology, University of California San Francisco, San Francisco, CA, USA; ^6^San Francisco General Hospital, San Francisco, CA, USA

## Abstract

*Background*. Low-income minority women with prior gestational diabetes mellitus (pGDM) or high BMIs have increased risk for chronic illnesses postpartum. Although the Diabetes Prevention Program (DPP) provides an evidence-based model for reducing diabetes risk, few community-based interventions have adapted this program for pGDM women.* Methods*. STAR MAMA is an ongoing randomized control trial (RCT) evaluating a hybrid HIT/Health Coaching DPP-based 20-week postpartum program for diabetes prevention compared with education from written materials at baseline. Eligibility includes women 18–39 years old, ≥32 weeks pregnant, and GDM or BMI > 25. Clinic- and community-based recruitment in San Francisco and Sonoma Counties targets 180 women. Sociodemographic and health coaching data from a preliminary sample are presented.* Results*. Most of the 86 women included to date (88%) have GDM, 80% were identified as Hispanic/Latina, 78% have migrant status, and most are Spanish-speaking. Women receiving the intervention indicate high engagement, with 86% answering 1+ calls. Health coaching callbacks last an average of 9 minutes with range of topics discussed. Case studies presented convey a range of emotional, instrumental, and health literacy-related supports offered by health coaches.* Discussion*. The DPP-adapted HIT/health coaching model highlights the possibility and challenge of delivering DPP content to postpartum women in community settings. This trial is registered with ClinicalTrials.gov NCT02240420.

## 1. Introduction

Following pregnancy, low-income, minority women with a history of GDM or high BMIs are at high risk for chronic illnesses, particularly obesity, type 2 diabetes mellitus (DM), and postpartum depression [1, 2]. Racial/ethnic disparities exist across a variety of postpartum health outcomes, and these disparities may be widened by less postpartum clinical follow-up by Hispanic/Latina and African American patients [3]. These disparities include a higher prevalence of gestational diabetes (GDM) during pregnancy, an increased risk for DM postpartum [4, 5], and a lack of postpartum diabetes screening for those with GDM. Only one in five Latina women with prior GDM returns for postpartum diabetes checkups, the lowest follow-up frequency of any group [6]. These women are lost to follow-up despite recommendations for six-week postpartum screening as well as annual diabetes checks [7].

In California, the prevalence of GDM is 5.7% among Latina women, compared to 4% in non-Hispanic whites [8, 9]. Women born outside the US make up a majority of pregnancies in California and may be at higher risk for GDM and subsequent DM for reasons including disruption to their normal eating habits, unhealthy dietary acculturation, barriers to physical activity, and rapid weight gain after migration to high-income countries [10, 11]. Results from the Diabetes Prevention Program (DPP) suggest that the long-term risk for diabetes postpartum can be reduced through behavior change [12–14]. Current strategies to expand postpartum care management and to reduce chronic disease risk for ethnic minority women include tailored interventions that address health literacy and provide accessible resources or health coaching on lifestyle changes, but there are few programs that focus on delivering the DPP content for early postpartum women in the period immediately following birth.

Health information technology (HIT) can be an important tool to tailor health communication efforts, and in particular if developed with end-users, it can be effective with low-literacy and low-income populations [15–19]. A review of literacy-focused interventions shows that interventions using health information technologies have high potential to reach low-literacy, high risk populations since they have flexibility of access in the home and during convenient times [20, 21]. Interventions providing longitudinal care and support through health coaching or counseling have also been effective in reducing chronic illness [22].

Approximately 85% of adults in the US are cell phone users, and cell phone use does not vary by race or ethnicity. Majority of low-income users have basic cell phones used for voice messaging and texting, and while this type of phone usage does not have the functionality of a smart phone (Web browsing and mobile applications), it provides access to the broadest range of users across SES status [16]. To improve women's health postpartum, several HIT models are effective in enabling communication and reaching women when the clinic-based model is less convenient. Postpartum low-income women may lose Medi-Cal eligibility, encounter barriers in accessing health care, and face new demands at home. Postpartum calls can reinforce messages received in health care encounters as well as facilitate uptake of preventive services for both mother and child/children in the 6–9 months after delivery (e.g., 6-week glucose testing after delivery and vaccinations in infants). In the case of diabetes, there is growing literature indicating that diabetes self-management support can be improved with improvements in patient satisfaction as well as diabetes-specific health outcomes through health technology (Interactive Voice Messaging; Internet-Based Systems). Our team has used a tailored combination of short (4-5 minutes) automated calls with queries and narratives in diabetes self-management and have found such an approach, Automated Telephone Self-Management Support (ATSM), effective for reaching and engaging patients with low health literacy and limited English proficiency and it can be cost effective and can improve health outcomes [23–25].

Another proven strategy for diabetes prevention is through health coaching. Health coaching involves counseling patients with chronic conditions to improve their own health by increasing their knowledge, skills, and confidence in managing their own health behaviors [26]. The effectiveness of health coaching in motivating, empowering, and enabling patients to improve health behaviors is now well established [27–31]. In particular, the peer health coach model has been successful in helping patients self-manage diabetes [32, 33]. Goldman et al. describe successfully using peer coaches, who were diabetic patients themselves, to provide support to other patients through three key roles: advisor, supporter, and role model [34]. Another study showed that the peer coach model is particularly effective among patients with worse medication adherence and higher HbA1c levels [35]. Some evidence suggests no difference in patient outcomes when comparing peer coaches and health professionals in the counsel of diabetes patients [36]. Heisler et al. randomized patients with diabetes to a diabetes care group managed by a nurse practitioner either with a peer coach or with a nurse practitioner alone and found that participants with the peer coach had greater improvements in HbA1c levels after intervention [30].

Despite the success of peer health coaching programs in primary care and clinical settings, there are few examples of similar programs implemented in community-based settings, such as federally funded programs or nonprofit organizations. There is also limited data on how such a model might address language and literacy challenges faced by low-income women with recent histories of migration to the US. One well-known and relevant community program that offers health coaching for this population is the Women, Infants, and Children (WIC) Program, which provides supplemental foods, health care referrals, and nutrition education for low-income pregnant and postpartum women [37]. In some WIC locations, peer-supported health coaching is available for breastfeeding support [38].

In this paper, we present a unique health coaching model, the STAR MAMA program (support via Telephone Advice and Resources/Sistema Teléfonico de Apoyo y Recursos-MAMA), which combines HIT-based queries and narratives, with follow-up by trained health coaches, to deliver adapted DPP content. The goals of this paper are to (1) illustrate a unique model of health coaching for high risk, low-income postpartum women which has relevance in community and clinic-based systems and (2) present case studies of exemplary STAR MAMA health coached participants stories.

## 2. Methods

STAR MAMA is an ongoing randomized clinical trial comparing a HIT-based health coaching program with a usual care arm providing an educational resource guide covering approaches to improving postpartum diabetes risk behaviors among GDM women based on DPP content. The study partnered with four key community sites: Zuckerberg San Francisco General (ZSFG), Santa Rosa Community Health Centers (Vista and Lombardi clinics) in Sonoma County, and San Francisco and Sonoma WIC offices. We chose to partner with WIC, as it is one of the few programs that provide services to low-income women spanning from pregnancy into the postpartum period and because migrant women can access most services on behalf of themselves or their infant. Additionally, WIC's program content, which provides information and resources on healthy eating and referrals to health care, aligns with the main objectives of the STAR MAMA program [37].

### 2.1. STAR MAMA Study Design and Evaluation

The STAR MAMA program is evaluated through a randomized control trial design in which women are assigned to one of two arms: (1) HIT arm: participants receive weekly phone calls from the automated telemedicine self-support system on various diabetes preventive topics and are matched to a health coach for longitudinal follow-up (2); usual care/education arm: participants receive an education resource guide (information and links to nearby resources) about postpartum care for themselves and their baby along with community resources for diet, physical activity, and so forth. Recruitment at clinic sites was monitored through a thorough list of GDM women who were approached based on eligibility criteria to enroll in the study. For the WIC sites, women were targeted based on delivery dates and either having GDM or a high BMI status (>25). WIC staff recruited women through phone calls prior to enrollment in the study. Women were randomized prior to conducting the baseline survey at their enrollment visit to one of the two study arms (using an envelope sealed randomization assignment), following administration of the baseline survey. Primary outcomes include self-reported weight, body mass index (BMI) based on chart review, receipt of recommended postpartum glucose testing, changes in dietary patterns, such as consumption of fruits and vegetables and foods high in fats or sugars, and physical activity (minutes per week). Prior to enrollment, all women completed a written or verbal consent.

### 2.2. STAR MAMA: Intervention Description

#### 2.2.1. STAR MAMA HIT Component

**Figure 1:**
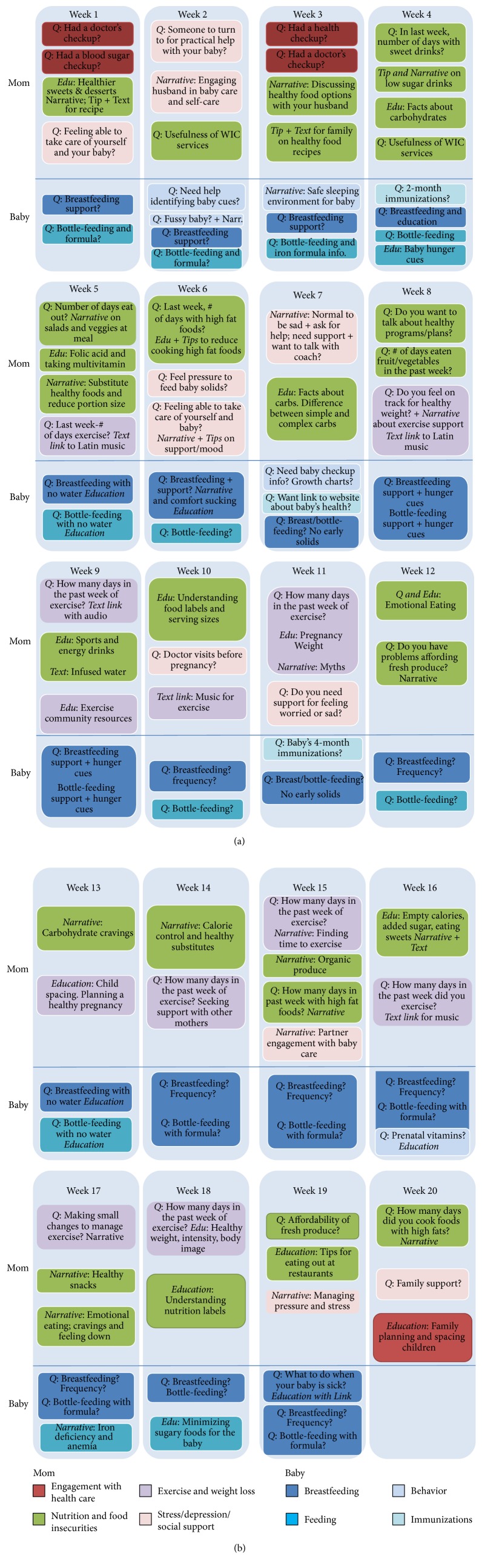
STAR MAMA HIT automated telephone messages content and mode of delivery: Maternal and Child Information (Edu), Queries (Q), Narrative, or Tip/Text.

*STAR MAMA *participants randomized to the intervention arm of the study receive weekly phone calls through our automated telemedicine self-support system and are paired with a health coach for longitudinal follow-up. The phone calls start 6 weeks postpartum and continue for 20 weeks. A HIT enabled model was selected at the outset because it allows participants to receive weekly content and health coaching support in their primary language while remaining in their homes, as traveling to appointments and group sessions is known to be a key barrier to receiving preventive services in community settings [39]. The calls focus on DPP topics including diet, physical activity, encouraging partner support, balancing self-care postpartum, healthy eating tips, importance of receiving a blood sugar checkup, and baby care. Information was delivered through recorded narratives and text tips in the automated telephone system. Women also received information from “live” health coach call backs [40]. For example, if a participant pressed “1” (yes) to a query asking if she was feeling stressed about her baby crying, she might hear a story about a new mom like her, facing similar challenges, reassuring her that it is ok to ask for help. The information from this question would then be delivered to her STAR MAMA health coach, who would also call her back and provide her with support.  Figure 1 describes the content and method of delivery of the range of STAR MAMA topics covered from weeks 1 through 20.

An enrolled participant can trigger a response several times during each phone call. Triggers are classified by predetermined values which determine whether a health coach callback is required. Daily and weekly reports from the HIT calls provide context for the health coaching call and motivational interviewing and help the coach understand what issues to focus on when developing an action plan or goals with the participant.

#### 2.2.2. STAR MAMA Health Coaching Component

A participant receiving the weekly phone calls is also matched to a health coach who monitors her response to the calls and regularly follows up with the participant on relevant issues. Follow-up topics vary from specific concerns, longitudinal support, empowerment, or resources for the mother. For example, in the sixth week of calls, the participant is queried:* “If you have questions about feeding your baby or how to deal with the pressure you are feeling from your family and friends, press 1 and a health coach will call you back. If not, press 2.”* Health coaches at ZSFGH were from bicultural backgrounds and had previous experience in the clinical setting as either health coaches or para-health professionals. Coaches at the WIC sites were trained nutritionists or registered dieticians already working in the WIC system. At minimum, each health coach received a two-day training at the University of California San Francisco (UCSF) Center for Excellence in Primary Care and was given a one-day STAR MAMA specific training that focused on delivery of the DPP, for a total of three days of health coaching training. Ongoing health coaching training also included review of the health coaching manual adapted from the DPP and biweekly review of cases at staff meetings. Additionally, coaches had ongoing meetings to discuss and reflect on common topics and support each other in the coaching process. Health coaches kept detailed notes based on call summary and trigger reports, which all coaches could access for internal resources. The health coaching curriculum was codeveloped with relevant stakeholders, including participants and care providers familiar with the ethnic diversity of the local populations. The following list titled “Health Coaching Training Topics, Example Query, Narrative, and Health Coach Script” describes a summary of the health coaching training, an example of a STAR MAMA automated telephone call, and a guideline for the subsequent health coaching topics.

*Health Coaching Training Topics, Example Query, Narrative, and Health Coach Script*

*Health Coach Training Topics*

- Communication
  - Ask-tell-ask, how to receive information from the participant
  - Closing the loop
  - Setting the agenda
  - Understanding your current health status, numbers
- Motivational interviewing
  - Exploring patients motivations and barriers
- Postpartum risk specific knowledge
  - Knowledge regarding risk of DM postpartum
- Community and clinic specific resources
  - Community related risks: safety, access to affordable produce, and primary care
  - Community-based resources: food banks, WIC agencies, free health clinics, and physical activity groups

*Health Coaching Example*

*Query to Participant*. “In the last 7 days, how many days did you drink sweetened drinks like sodas, aguas frescas, fruit juices, coffee with sugar or condensed milk, sports drinks or energy drinks? Press the number of days.”

*Callback Trigger. *Callback trigger ≥3 days (this trigger is determined by the participant pressing 3–9 on her phone, as a response to the question about the number of days she drank sugar sweetened drinks).

*Narrative, Heard by Participant*. “*Sweetened drinks taste good but don't have healthy calories. A small can of soda can have as many as 10 sugar packets in it! Even aguas frescas can have extra calories and loads of sugar without giving you much nutrition. You don't have to stop drinking them, but you can try having them less often and making your own with less sugar. First try preparing them with half the amount of sugar. For example, if you like preparing agua fresca with strawberries and usually add two large spoonfuls of sugar, try just adding one. At first it might taste less sweet but it is something that you and your family can get used to, and is part of showing them your commitment to health- theirs and yours!*”

*Health Coach Script and Topic Guide*

*Verify Report*

- “On your call you answered that in the past 7 days you drank sweetened drinks ____ days. Is that correct?”

*Open-Ended Question*

- “How many sugary drinks do you typically have in one day?”
- “What do you like about drinking sweetened drinks?”
- “Is there something you don't like about drinking them?”
- “How do you think drinking sweetened drinks affects your health?”
- “How do you think it could affect your risk of developing T2DM?”

*Provide Education*

- Sugary drinks contribute to high calorie intake and can lead to obesity.
- Drinks high in sugar make your blood sugar spike within a few minutes of drinking it.
- People who consume sugary drinks regularly—1 to 2 cans a day or more—have a greater risk of developing type 2 diabetes than people who rarely have such drinks.

*Help Participant Make Action Plan*

- “What step would you like to take to start reducing your intake of sugary drinks?”
- “When are you going to do it?”
- “How much are you going to decrease them?”
- “How often are you going to do it?”
- “On a scale of 1–10, 1 being not sure at all and 10 being completely sure, how sure are you that you can _______ by ______?”

If less than 7, encourage participant to modify action plan to make it achievable.

**Figure 2:**
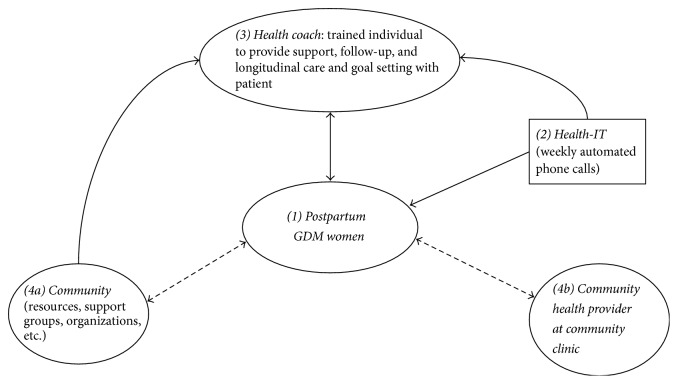
STAR MAMA health-IT intervention linkage model: using the health coach as a bridge between the community and hospital infrastructure for postpartum GDM women. (1) A woman is enrolled into the STAR MAMA study based on her eligibility. See Table 1 for baseline demographics. Eligible WIC participants were referred to the STAR MAMA study by their respective coordinators. (2) Enrolled participants select call times to receive proactive calls or call in toll-free from the automated telemedicine system. Each week participants receive a rotating set of prevention-focused queries, narratives, and texts (e.g., on diet, exercise, breastfeeding, and baby care). If a participant enters a value predefined as “out of range,” participants also hear recorded first person supportive narratives related to their “out-of-range” reply encouraging behavior change as well as narratives offering shorter tips. (3) Each participant is matched with a health coach, a trained nonprofessional individual recruited for this study. The health coach is trained on health coaching protocol and diabetes prevention (Center for Excellence in Primary Care). The coach receives automatically downloaded daily reports from the ATSM calls and participant responses. Depending on the participant's needs, the health coach calls back to provide participant with emotional support and engage participant in goal setting/action and provides information about community resources. (4) ((4a) and (4b)) The health coach can connect the patient with community programs, food banks, farmers markets, WIC counselors, mental health support groups, and so forth. Additionally, the coach may send a notification to a patient's clinic and/or clinician if deemed urgent, based on predetermined protocols.

While the automated phone calls provide participants with passive information and support through narratives, the health coaches directly reach participants, explore their needs, build on their strengths, and set goals to help them reach their health targets. As such, the health coach serves as a bridge between the participant and the primary care clinic and as a source of support, resources, and accountability.  Figure 2 illustrates the relationship between the health coach, the ATSM service, primary care providers, the community, and the participant within the STAR MAMA study. In this patient-centered model, the health coach is an integral source of tangible preventive information and longitudinal communication with the participant and health care setting, community, or clinic.

### 2.3. STAR MAMA Participants

This paper includes a sample of women who have either completed STAR MAMA or are currently enrolled representing half of the targeted recruitment sample. Eligibility criteria for STAR MAMA include 18–39 years of age, at least 32 weeks pregnant, and either a GDM diagnosis or BMI > 25. Participants were recruited from our four community sites through either physician referral, WIC referral, or direct communication during scheduled prenatal appointments.

### 2.4. Assessment of Engagement with STAR MAMA Program

Participant engagement was assessed using our online database tracking system, which monitors participant weekly call responses. Different levels of engagement were determined based on measures in previous studies: (1) participant had completed at least one of the weekly phone calls; (2) average number of calls completed out of the 20 weeks of calls, and (3) among those completing one or more call, the percentage of calls completed over the intervention period [26].

All women receive a baseline visit, 3-month short phone survey and a 9-month postpartum follow-up survey and will have their medical charts reviewed over the study period. The follow-up surveys are used to assess feasibility, acceptability, and health related outcomes (e.g., weight loss, physical activity, consumption of healthy foods, breastfeeding, replacement of water for sugar sweetened drinks, and glucose screening). For the women enrolled in the health-IT arm, engagement is tracked through the HIT system, in which we monitor the calls women are responding to and the queries they trigger for. A health coach also monitors the participant's progress through extended phone calls for resources and support.

To achieve empirical results regarding the success of the program, key stakeholders in our partner sites were consulted to iteratively assess the implementation of the program. A group of regional and national advisors was assembled to help assess the challenges in integrating the STAR MAMA model and to critically evaluate the feasibility and acceptability of this hybrid HIT and health coaching model in the community setting. The primary advisors were from WIC, and they included research staff, management, and nutritionists. Based on discussions, reflections, and informant interviews with our advisors and partners, we were able to articulate key barriers to our model and assess the scope of scalability.

### 2.5. Selection of Case Studies

Case studies were selected from WIC sites to represent health coaching calls conducted in community settings. Two participants from each the San Francisco (SF) and Sonoma County WIC sites were chosen to reflect the diversity of women's experiences and the diversity of coaching content.

## 3. Results

### 3.1. Participant Sociodemographic Characteristics

Table 1 describes key demographic characteristics of the study population (*N* = 86). Women enrolled were on average 30 years old; 78% were identified as Latina or Hispanic and were not born in the US. Migrant women lived an average of 10 years in the US and 63% listed Spanish as their preferred language. Of the subsample, 23% were members of the WIC program. The women had, on average, two children below the age of 18 currently living in their household. Eighty-seven percent of women were diagnosed with gestational diabetes previously, of which 97% were diagnosed during their most recent pregnancy. Thirty-six percent were obese, overweight, or experience unhealthy weight gain during their pregnancy.

### 3.2. STAR MAMA Engagement

Since the STAR MAMA program enrollment is ongoing, engagement data is representative of the first wave of enrollment, accounting for almost half of the recruitment target. Among the study subsample (*N* = 86), twenty-eight women have completed all 20 weeks of the HIT and health coaching program. Of those women, 89% answered at least 1 phone call, with an average of 12 total phone calls answered out of 20 weekly calls. On average, the women answered 61% of calls during the intervention period. Among all women randomized to the STAR MAMA HIT and health coaching arm (*n* = 43), excluding those who withdrew from the study or are lost to follow-up, 86% have answered at least one phone call till date (Figure 4).

### 3.3. Case Studies from San Francisco WIC and Sonoma County WIC

Table 2 presents four case studies from SF and Sonoma country WIC sites. The women receiving the STAR MAMA HIT program through weekly calls were at high risk and triggered for poor physical activity, high carbohydrate, fat or sugar consumption, signs of depression of feeling “overwhelmed,” and more. The length of the health coaching callbacks ranged from short follow-ups of 3 minutes to up to 45 minutes in some cases, with an average of 9 minutes per call. Topics covered ranged from the health-IT phone call narrative topics (diet control, physical activity, depression, cutting back on high fat and sugary foods, breastfeeding, and bottle-feeding) to miscellaneous health needs of the participants. In Table 2, we describe synopses of coaching calls with relevant actionable items, such as follow-up topics, and community or clinic implications.

**Figure 3:**
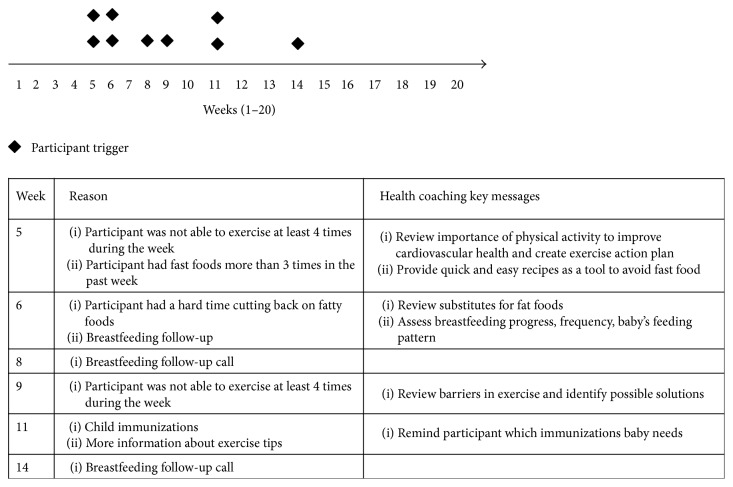
HIT enabled phone call system, participant triggers, and context-based health coaching messages: summary for Ms. C. at San Francisco WIC. Timeline of calls and weekly triggers indicated by Ms. C. The timeline displays the weekly phone calls to Ms. C., from weeks 1 through 20 by the ATSM system. The diamonds indicate triggers and actionable events, and the table summarizes the reason for triggers each week. A health coach monitors the daily and weekly reports from the HIT system to follow-up with the participants through a trigger based approach.

**Figure 4:**
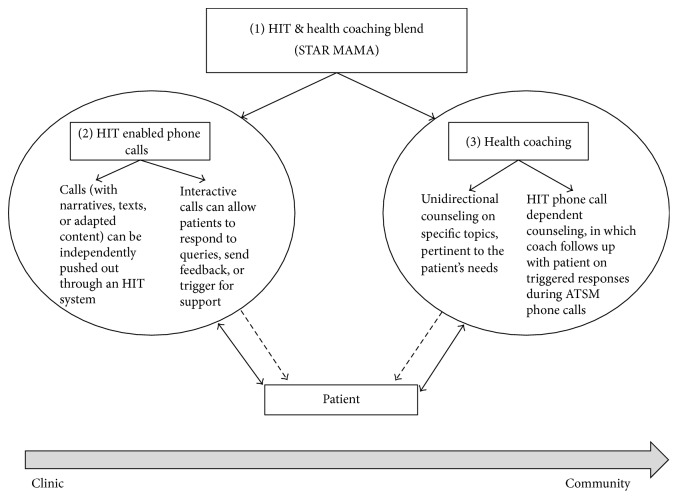
Multimodal adaptation of the STAR MAMA HIT/health coaching hybrid model to meet community needs. This model breaks down the different modes of implementation of the STAR MAMA model to illustrate the flexibility of supporting self-managed care within the clinic to community spectrum. Both components of the model, HIT and health coaching, have the capacity to interact uni- or bidirectionally with the patient in the clinic or community setting. (1) STAR MAMA: the STAR MAMA intervention is a blend of weekly HIT phone calls to eligible patients and health coaching calls for support and follow-up. (2) ATSM: this is one component of the STAR MAMA model, in which patients receive weekly phone calls for 20 weeks on various topics regarding postpartum health. The calls can be implemented in the community setting unidirectionally, in which the patient listens to educational narratives, or the phone calls can be programmed to offer an interactive component. (3) Health coaching: trained health coaches can provide topic-based counseling to patients regarding specific topics tailored to the patient's needs. Or the health coach can receive triggers from an HIT system (if both are used in conjunction) to follow-up with patient on high risk issues.

These stories illustrate accounts of coaching to women who are representative of the enrolled participants in the STAR MAMA program. It is evident that the health coach serves as a connection between the woman postpartum and critical resources, including information, basic knowledge and tips about postpartum care, and links to their preferred primary care system. Moreover, the health coach is a key point of support not only for the health-IT call-based topics, but also for miscellaneous questions the new mother has doubts about.  Figure 3 demonstrates how the HIT component integrates with health coaching and how the coaches use the participant-driven triggers to direct coaching calls and discuss specific and relevant topics during a session.

These vignettes illustrate the depth and breadth of issues covered by health coaches during their interactions with participants. They highlight major themes and barriers to self-management postpartum including (but not limited to) need for improved resources for child care after delivery, reinforcement for reduction of sugar and fat consumption, goal setting and action planning to improve physical activity, reminders about the importance of follow-up blood sugar testing postpartum and postpartum depression screening.

## 4. Discussion

Because high risk and low-income postpartum women often do not receive the longitudinal care and support they need to reduce their risk of DM and other chronic illnesses, this is a critical window of opportunity to intervene and provide maximal resources, support, and tools for prevention of chronic illnesses such as diabetes and also help self-manage existing chronic conditions. In this paper, the example coaching calls suggest that health coaches in STAR MAMA act as a bridge between a participant and the primary care system to emulate a continuum of care even after delivery.

Scalable implementation of health coaching, a HIT enabled model, or a hybrid HIT and coaching model can have community and clinic specific benefits but there are several identified barriers to such an attempt at integration. We investigated these potential barriers with community programs through discussions with our regional advisors at San Francisco and Sonoma WIC. In the following, we articulate three core limitations to health coaching specifically identified within this specific community setting.

First, community WIC programs have boundaries regarding the services they can offer and may face restrictive funding for programs like health coaching and low capacity to train and hire coaches. In the context of WIC, while there is a currently funded and high functioning Peer Coaching program (Loving Support Peer Counseling) it focuses primarily on breastfeeding and lactation support. As such, peer coaches within the WIC infrastructure are not trained to coach women on critical postpartum topics, such as diet, physical activity, postpartum depression, healthy eating tips, and family support. Moreover, not all counties within states have the funding allocated for the Loving Support Peer Counseling Program, and absorbing a HIT or health coaching component can strain their budget.

A second major barrier for health coaching in community settings is the limited availability of coaches who have expertise required within the program structures. For example, within WIC, those who provide nutrition counseling are often expert health professionals: dieticians, nurses, or trained diabetes educators. In most sites, though there are few of these expert health professionals available to receive additional training as health coaches for a broader set of concerns outside of nutrition. On the other hand there are often peer coaches, who are more numerous but less well trained, and they are not able to coach beyond a more limited scope, as with an emphasis on breastfeeding and lactation.

Lastly, a major limitation in prevention in such high risk, low-income populations is that they are very hard to reach and follow-up with [16, 41]. Women who are at most risk for chronic illnesses like type 2 diabetes, obesity, or depression postpartum are often from the most vulnerable populations, who historically have transient housing situations and may have difficulty engaging in such a program. Even with a HIT blended approach such as STAR MAMA, where a health coach counsels patients over the phone, reaching patients is the biggest barrier in coaching. In particular, women who start working after delivery are the most difficult to contact, with their irregular and often hectic schedules. However, the period after delivery is a very critical time, when women require the most support to adjust to their physical and mental changes after having a baby.

## 5. Conclusion

It is critical to consider the positive impacts of health coaching and health-IT interventions in the clinical settings and develop techniques to execute these strategies in the community. Our preliminary conversations with key stakeholders (County WIC staff, STAR MAMA health coaches, and National WIC advisors) have outlined the scope of integration of these interventions in the community settings and have addressed the need to expand care through methods like telemedicine. A model like STAR MAMA may be daunting to implement within a community structure; particularly when funding is limited, scopes of practice are restricted, or participants are hard to reach. However, frameworks of self-care management and behavior change using HIT, health coaching, or both will be extremely beneficial in the future to improve preventive health practices in communities and potentially mitigate the disease burden that a safety net hospital or community clinic may face. We need more innovative programs to bridge counseling and resources between the patient and provider, facilitated by health information technology. While challenges persist, the flexibility of these interventions and evidence-based success in the clinical setting urges expanding care to community programs.

## Figures and Tables

**Table 1:** Sociodemographic characteristics of currently enrolled or completed STAR MAMA participants (*N* = 86).

	Both study arms (education resources and HIT arm) (*N* = 86) *N*%
Age (in years), mean (SD)	30.05 (5.16)
Race/ethnicity, *n* (%)	
Asian or Pacific Islander	7 (8.2%)
Black or African American	6 (7.1%)
White or Caucasian	4 (4.7%)
Latino (a) or Hispanic	67 (78.8%)
Others	1 (1.2%)
Children currently in household under 18 years of age, mean (SD)	1.68 (1.31)
Born outside US, *n* (%)	66 (77.6%)
If not born in US, total years living in US, mean (SD)	10.27 (6.57)
Previously diagnosed with gestational diabetes, *n* (%)	72 (86.7%)
Of those with GDM, diagnosed during this pregnancy, *n* (%)	67 (97.1%)
Previously diagnosed with overweight, obese, or unhealthy weight gain, *n* (%)	30 (36.1%)
Of those overweight, obese, or unhealthy weight gain, diagnosed during this pregnancy, *n* (%)	10 (71.4%)
Preferred language, *n* (%)	
English	32 (37.2%)
Spanish	54 (62.8%)
WIC status, *n* (%)	
Non-WIC	66 (76.7%)
WIC	20 (23.3%)

**Table 2:** Health coaching case studies: San Francisco and Sonoma WIC participants.

Site	Case studies: summaries of coaching calls with women enrolled in the HIT and coaching arm	Health coaching actions	Examples of range of efforts undertaken to address complex emotional, health literacy, and instrumental needs
San Francisco WIC	(1) Ms. C., a 33-year-old, Latina woman with a recent history of diet-controlled GDM received her first health coach call during week 1 of the STAR MAMA study, when she was 8 weeks postpartum. She is a homemaker and not married, but living with a partner in a marriage-like relationship. Ms. C. delivered her baby at 39 weeks. While she understood key steps and requirements for baby care, she reported feeling overwhelmed due to the stress of caring for her three other children as well. In addition to reported practical support and feeling like she had someone to listen to her; the health coach provided support and identified that she was not suffering from depression. In the third week, Ms. C. reported mixed-feeding for her baby with breast milk and pumped milk, a shift from her exclusive breastfeeding in the past two weeks. Her health coach encouraged her to exclusively breastfeed whenever possible and reviewed the importance of breast milk for a growing infant. With this and other supports, Ms. C., who reported high intention to breastfeed prior to pregnancy, was able to eventually continue without formula for the first 6 months. During the fifth week, Ms. C. reported binging on unhealthy snacks: soda, sweets, and foods from the local taqueria. After querying about her symptoms, her health coach was concerned that she displayed signs of elevated blood sugar. She discussed the dangers of a high fat and high sugar diet and encouraged her to replace soda with water. Together, they set goals and her health coach followed up weekly to assess her implementation of her action plan. Additionally, her coach helped Ms. C. make an appointment with primary care provider to get her blood sugar rechecked	(i) Supportive counseling and provision of postpartum stress management strategies (ii) Encouragement of sustained breast feeding for first 6 months and instruction for safe bottle-feeding (iii) Assessment of participant-specific barriers to reducing a high sugar and high fat diet; develop a set of resources and tools to share with participant for managing postpartum eating habits for self and family (iv) Assistance with reconnecting with the primary care setting (community clinic or hospital) to follow-up on her health status	(i) Participant was uncertain about the meaning of her screening test result and what to do next (ii) Participant benefited from tailored breast feeding support within the context of the stresses encountered with multiple children's needs to address besides baby

San Francisco WIC	(2) Ms. F., a 21-year-old Latina woman with a recent history of diet-controlled GDM during her pregnancy. During pregnancy she worked part-time (<20 hours) in food delivery and was not married but living with only her partner and pregnant with her first child. She received her first health coaching call during her week 1 of enrollment in STAR MAMA, 8 weeks postpartum, when she reported feeling like she could not do all the things she needed for her baby. She was occupied with her baby's belly button, which she thought looked a bit abnormal, and was denied a follow-up appointment since the baby's MediCal was inactive. Her health coach informed Ms. F. about the different MediCal managed care plans and advised her on how to communicate with MediCal and switch her baby to a good plan. During pregnancy, Ms. F. reported high intention to breastfeed and mainly breastfed her baby, supplemented sometimes with formula. Her health coach reinforced the importance of exclusive breastfeeding and offered a breast pump from WIC for Ms. F. to borrow. In the following weeks, Ms. F. contacted MediCal and was able to get her case reviewed. Though she wanted to start her baby on solid foods, her health coach suggested waiting until the baby was approaching 5-6 months and she reviewed the risks of starting solid foods preemptively. Ms. F. was motivated to follow these recommendations and take full precaution when feeding her baby. She also cleared her doubts about babies burping and fat consumption with her health coach	(i) Provide support and knowledge on postpartum baby care and time management (ii) Guide participant with activation and follow-up of baby's MediCal plan (iii) Review and instruct participant on proper complementary feeding practices (timing, what to start, frequency, assessing hunger cues) (iv) Answer miscellaneous questions and provide longitudinal support on various doubts (belly button, baby burping, etc.) that participant may have as a new mother	(i) Participant needed support and guidance on how to renew her baby's MediCal plan to facilitate continued care (ii) Participant received extra support and resources from the health coach (i.e., breast pump from WIC) to enable exclusive breastfeeding and instructions on how and when to start complementary feeding

Sonoma County WIC	(3) Ms. H., a 33-year-old Latina woman was enrolled in the study at 7 weeks postpartum and received health coaching calls during her fifth week in the study. She is a homemaker and married and delivered her fourth child at full-term. Her health coach gave her ideas about encouraging children to eat healthier foods, that is, fruits and vegetables, since she had younger children in her family (two children <5 years old, in addition to the new baby) who were fussy eaters. She was not too keen on exercising, but her health coach applauded her for trying at least once a week and encouraged her to exercise more frequently which was a great accomplishment; though at home Ms. H. felt that she had someone to listen to and comfort her, she did not feel that she had support with practical help. During the 15th week, Ms. H. struggled with cutting back on fatty foods and sugary drinks, like sodas. Her health coach worked with her to make a plan and incorporate quick tips to address these issues, such as draining fat during cooking and making homemade agua frescas. Her health coach also provided her with many local, community-based food resources. Ms. H. was left feeling supported, motivated, and confident in her ability to make changes (see Figure 3)	(i) Provide tips on managing other children, especially encouraging healthy habits for fussy eaters (ii) Improve knowledge about exercise benefits and suggest recommendations and strategies to integrate physical activity into participant's daily schedule (iii) Discuss techniques to substitute high fat/sugar foods with healthier alternatives	(i) Participant needed information and support on how to control the diets of fussy eaters (ii) Participant needed reinforcement on exercise and healthier substitutes (iii) Participant received relevant information on local food banks and community resources to improve self-management and care

Sonoma County WIC	(4) Ms. G. is a 24-year-old Latina woman. She is a homemaker, living with a partner in a large household of 12 individuals. Though she reports feeling like once she had the baby she was able to get help with cooking and other tasks, she did not feel supported emotionally. Her baby was born full-term in a vaginal birth. She was enrolled into the study and started receiving health coaching calls at 7 weeks postpartum. Due to poor latching, Ms. G. breastfed for just 3 weeks and started with formula milk for her baby. Her health coach instructed her on safe bottle-feeding practices and reviewed hunger cues to prevent overfeeding and recognize signs of fullness. Around the sixth week, Ms. G. reported feeling overwhelmed, tired, and depressed. Her health coach discussed the commonness of baby blues after delivery and relevant symptoms and encouraged Ms. G. to speak with her primary care provider about this in her upcoming appointment. In follow-up calls, Ms. G. was able to appropriately recognize hunger cues and her mood improved after taking her multivitamins and eating properly	(i) Review safe bottle-feeding practices and support on adapting to baby hunger cues (ii) Provide emotional support and reassurance about baby blues (iii) Facilitate connection between participant and primary care provider postpartum to acknowledge issues of postpartum depression	(i) Participant received preliminary postpartum depression screening, so she could be appropriately directed to a primary care provider (ii) Participant was uncertain about baby hunger cues and proper bottle-feeding practices
